# Cushing’s syndrome caused by ACTH-producing thymic typical carcinoid with local invasion and regional lymph node metastasis: a case report

**DOI:** 10.1186/s40792-018-0459-7

**Published:** 2018-06-11

**Authors:** Wakako Fujiwara, Tomohiro Haruki, Yoshiteru Kidokoro, Takashi Ohno, Yohei Yurugi, Ken Miwa, Yuji Taniguchi, Hiroshige Nakamura

**Affiliations:** Division of General Thoracic Surgery, Department of Surgery, Faculty of Medicine, Tottori University, 36-1, Nishi-cho, Yonago, 683-8504 Japan

**Keywords:** Thymic carcinoid, ACTH, Cushing’s syndrome, Total thymectomy

## Abstract

**Background:**

Ectopic ACTH-producing thymic carcinoid tumors are rare, but often behave aggressively with local invasion and distant metastasis. We herein report a case of ACTH-producing thymic typical carcinoid tumor with lymph node metastasis treated by surgery and postoperative radiation therapy.

**Case presentation:**

A 61-year-old woman was admitted to be evaluated for hypoglycemia and hypokalemia. Laboratory data revealed elevation of serum cortisol and ACTH levels. Overnight administration of 8 mg dexamethasone did not suppress plasma ACTH. Chest CT demonstrated a tumor of 30 mm in diameter and enlargement of the lymph node at the anterior mediastinum. Ectopic ACTH syndrome was suspected and total thymectomy and lymph node dissection were performed. The histopathological examination indicated typical carcinoid tumor and mediastinal lymph node metastasis, and immunohistochemical staining was positive for ACTH. The plasma ACTH level decreased immediately after surgery. She received postoperative radiation therapy of 60 Gy.

**Conclusion:**

Ectopic ACTH-producing thymic typical carcinoid tumors are rare, and it is important to consider this disease and perform appropriate treatment.

## Background

Among adrenocorticotrophic hormone (ACTH)-dependent Cushing’s syndrome, 10–20% is due to nonpituitary tumors termed ectopic ACTH syndrome (EAS). The most common cause of EAS is small cell lung cancer, followed by thymic carcinoids. Thymic carcinoids are very rare neuroendocrine tumors that often complicate endocrine disorders. Although previously assumed to be variants of bronchopulmonary carcinoid tumors, they are generally more aggressive and difficult to treat. It is widely accepted that surgical resection is the only curative treatment for localized lesions, and the efficacy of chemotherapy and radiotherapy has not been well established.

We herein report a case of EAS caused due to a thymic typical carcinoid tumor successfully treated by surgery followed by radiation.

## Case presentation

A 61-year-old woman visited her primary care doctor because of general malaise, face edema, skin pigmentation, insomnia, and polyuria. Blood examination revealed marked hypokalemia and impaired glucose tolerance. Bilateral adrenal enlargement was observed on abdominal ultrasonography, and she was referred to our hospital for further examination. Endocrine examination showed both elevated plasma cortisol (107.7 pg/mL) and ACTH levels (1100 pg/mL), and increased urinary excretion of free cortisol (6650 mcg/day) and 17-ketogenic steroids (78.7 mg/day). Plasma cortisol and ACTH levels were elevated without any diurnal rhythm. Plasma cortisol was not suppressed by the overnight 8-mg dexamethasone suppression test. There was no response of plasma ACTH or cortisol to exogenous corticotropin-releasing hormone (CRH). Other hormones of the pituitary, thyroid, and adrenal medulla were all in normal ranges. Thus, ectopic ACTH syndrome was strongly suggested.

**Fig. 1:**
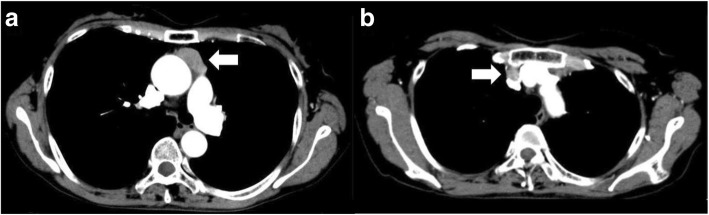
Chest CT image. A tumor (30 × 30 × 14 mm) without invasion localized in the anterior mediastinum (**a**). Enlargement of lymph node (**b**)

Chest computed tomography (CT) demonstrated a tumor of approximately 30 mm in diameter and enlargement of the lymph node in the anterior mediastinum (Fig. 1). High accumulation of 18-fluorodeoxyglucose in the anterior mediastinum tumor (maximum standardized uptake value [SUV] 2.48) but not in the lymph node was observed on positron emission tomography (PET)/CT. Somatostatin receptor scintigraphy also revealed mild uptake in the tumor. Collectively, these data were consistent with a diagnosis of EAS caused by an anterior mediastinum tumor, possibly thymic carcinoid tumor. There was no abnormal finding indicating multiple endocrine neoplasia (MEN).

**Fig. 2:**
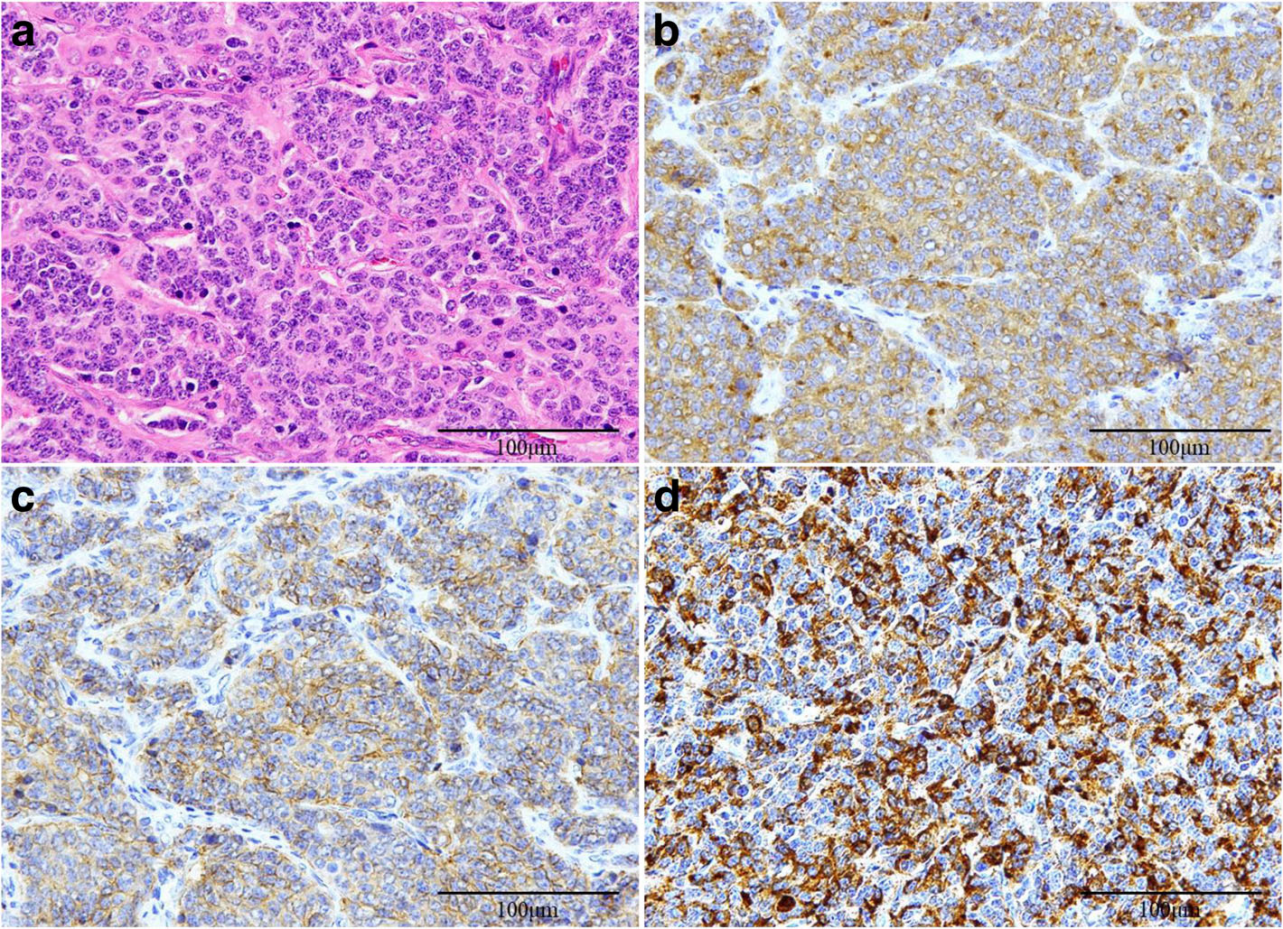
HE staining (**a**) indicated typical carcinoid tumor. Tumor cells were positive for synaptophysin (**b**), CD-56 (**c**), and ACTH (**d**) on immunostaining

**Fig. 3:**
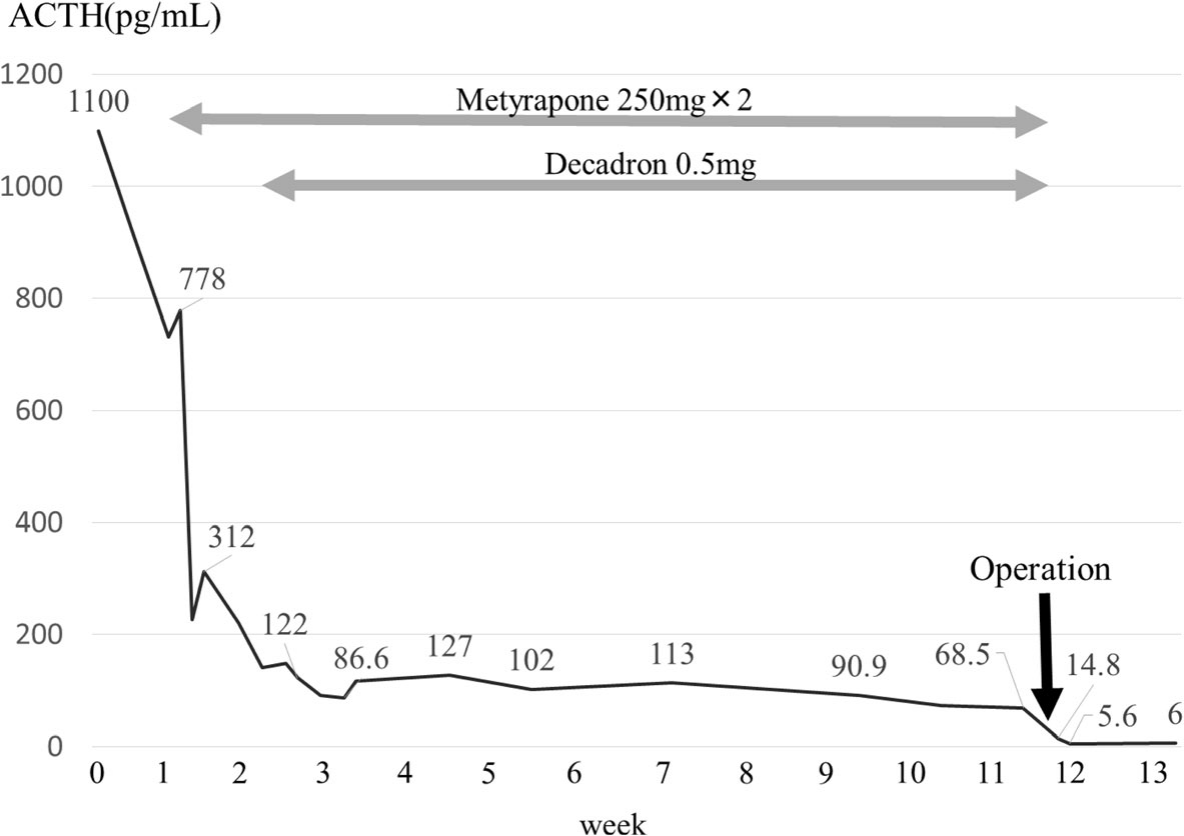
Changes in plasma ACTH levels during the clinical course

Before the operation, we administered 500 mg/day of metyrapone, and both ACTH and cortisol levels decreased to 68.5 pg/mL and 3.02 mcg/mL respectively. After 2 months of medical treatment, her symptoms were relieved and bilateral adrenal enlargement decreased. Under open thoracotomy by median sternotomy, she underwent total thymectomy, pericardial partial resection, dissection of the anterior regional and the right paratracheal lymph nodes, and sampling of the subcarinal lymph node. Histopathologically, the tumor consisted of round to spindle-shaped cells with high nucleus/cytoplasm ratios containing finely granular chromatin. Necrosis was absent, and mitotic figures were infrequent, with less than two per ten high-power fields (HPF). Tumor cells were positive for chromogranin A, synaptophysin, CD-56, and ACTH on immunohistochemistry (Fig. 2). The tumor had invaded the pericardium, and mediastinal lymph nodes were positive for metastasis. The final diagnosis was stage IVA (pT2N1M0) ACTH-producing thymic typical carcinoid tumor. The plasma ACTH level decreased to 14.8 pg/mL, less than normal, immediately after surgery (Fig. 3). Hydrocortisone was administered during the perioperative period and was gradually tapered, and finished 4 months after surgery. She received postoperative radiation therapy of 60 Gy. At 8 months after surgery, she showed no sign of Cushing’s syndrome or recurrence of the tumor without any medications.

## Discussion

Ectopic ACTH-producing thymic carcinoid tumor is an extremely rare clinical condition, comprising 29% of all thymic carcinoids and 5–42% of all ectopic ACTH-producing syndrome [1, 2]. It has been reported that radical surgical resection of the ACTH source is the only effective treatment [3]. Prior to surgery, medication therapy should be done to prevent perioperative complications and perform surgery when hormone values and symptoms are controlled. Furthermore, there is a risk of postoperative adrenal insufficiency; strict perioperative management is desirable.

Unlike pulmonary and other carcinoid tumors, thymic carcinoids often behave aggressively as an advanced disease with local invasion, lymph node metastasis, and distant metastasis because of the high proportion of atypical carcinoid tumors. Regarding ACTH-producing thymic tumors, Neary et al. reported three cases of well-differentiated ACTH-producing thymic neuroendocrine carcinomas, and the patients had no lymph node metastasis, recurrence, or death. On the other hand, nine cases of moderately differentiated ACTH-producing thymic neuroendocrine carcinomas almost had lymph node metastasis, and all patients had recurred [4]. However, our case was a typical carcinoid tumor with lymph node metastasis and local invasion.

As a surgical procedure, a median sternotomy approach is generally recommended because this enables excision of the entire thymus, perithymic fat, other affected mediastinal structure, and aggressive lymph node dissection. However, there is no standard for lymph node dissection in thymic epithelial tumors even though lymph node metastasis is an important prognostic factor. Hwang et al. recommended right paratracheal node dissection in addition to anterior regional lymph node dissection for TNM clinical stage II or higher diseases because they are crucial stations on the lymphatic pathway of thymic malignancies [5]. In the present case, we performed total thymectomy, followed by lymph node dissection of the anterior regional and right paratracheal nodes, and sampling of subcarinal lymph node via median sternotomy. The anterior mediastinal lymph nodes were positive for metastasis. Consequently, we considered the extent of lymph node dissection to be adequate, and radical resection was completed because the postoperative plasma ACTH level was successfully decreased. Although a good prognosis is expected by combined surgery and radiation, relatively high malignancy characteristics are observed compared with typical carcinoids, and strict follow-up is needed.

## Conclusion

We report a rare case of ectopic ACTH-producing thymic typical carcinoid with local invasion and regional lymph node metastasis. Surgical resection was effective to control Cushing’s syndrome in this case, and nodal staging may help to guide adjuvant treatment, but systemic nodal dissection/sampling is yet to be standardized.

## Abbreviations

ACTH: Adrenocorticotrophic hormone
CRH: Corticotropin-releasing hormone
CT: Computed tomography
SUV: Standardized uptake value
PET: Positron emission tomography
MEN: Multiple endocrine neoplasia
HPF: High-power fields
CD-56: Cluster of differentiation-56

## References

[1] Yoshikawa T, Noguchi Y, Matsukawa H (1994). Thymus carcinoid producing parathyroid hormone (PTH)-related protein: report of a case. Surg Today.

[2] Alexandraki KI, Grossman AB (2010). The ectopic ACTH syndrome. Rev Endocr Metab Disord.

[3] Zhou X, Hnag J, Che J (2016). Surgical treatment of ectopic adrenocorticotropic hormone syndrome with intra-thoracic tumor. J Thorac Dis.

[4] Neary NM, Lopez-Chavez A, Abel BS (2012). Neuroendocrine ACTH-producing tumor of the thymus—experience with 12 patients over 25 years. J Clin Endocrinol Metab.

[5] Hwang Y, Park IK, Park S (2016). Lymph node dissection in thymic malignancies: implication of the ITMIG lymph node map, TNM stage classification, and recommendations. J Thorac Oncol.

